# Fibromyalgia in pregnancy and postpartum period: effects on quality of life, obstetric outcomes, delivery decisions, and breastfeeding: a systematic review

**DOI:** 10.61622/rbgo/2026rbgo49

**Published:** 2026-08-20

**Authors:** Bruna Maria dos Santos Pereira, Vinícius Borges Alencar, Liana Maria Tôrres de Araújo Azi, Durval Campos Kraychete, Rodrigo Leal Alves

**Affiliations:** 1 Universidade Federal da Bahia Faculdade de Medicina da Bahia Salvador BA Brazil Faculdade de Medicina da Bahia, Universidade Federal da Bahia, Salvador, BA, Brazil.; 2 Universidade Federal da Bahia Faculdade de Medicina da Bahia Departamento de Anestesia e Cirurgia Salvador BA Brazil Departamento de Anestesia e Cirurgia, Faculdade de Medicina da Bahia, Universidade Federal da Bahia, Salvador, BA, Brazil.

**Keywords:** Breast feeding, Fibromyalgia, Pregnancy, Postpartum period, Quality of life

## Abstract

**Objective:**

To systematically evaluate the impact of fibromyalgia (FM) during pregnancy and the postpartum period on health-related quality of life (*HRQoL*), obstetric outcomes, and breastfeeding practices.

**Data Sources:**

PubMed/MEDLINE, EMBASE, Cochrane CENTRAL, and SciELO were searched for studies published between January 2010 and May 2024. The review followed Cochrane Collaboration standards and PRISMA 2020 recommendations.

**Study Selection:**

Randomized and observational studies assessing FM during pregnancy or postpartum were eligible. Titles, abstracts, and full texts were independently screened by two reviewers according to predefined inclusion and exclusion criteria.

**Data Collection:**

Data were extracted on maternal HRQoL, psychological well-being, obstetric complications, and breastfeeding outcomes. Methodological quality was appraised using ROBINS-I for non-randomized studies.

**Data Synthesis:**

From 152 records identified, five studies met inclusion criteria, encompassing 8,182 women with FM. FM was associated with greater pain intensity, reduced functional capacity, lower HRQoL, and higher rates of depressive and anxiety symptoms. A population-based cohort showed modestly increased risks of gestational diabetes, preterm birth, and cesarean delivery, while reproductive capacity and breastfeeding duration appeared unaffected. Most studies presented moderate risk of bias due to confounding and reliance on self-reported data.

**Conclusion:**

FM during pregnancy appears to be associated with reduced maternal HRQoL and impaired psychological well-being and may be associated with a modest increase in obstetric risk.

**PROSPERO registry:** #CRD42024572007

## Introduction

Fibromyalgia (FM) is a chronic pain syndrome characterized by widespread musculoskeletal pain, fatigue, sleep disturbances, and cognitive dysfunction, attributed to mechanisms of central sensitization and neuroendocrine dysregulation.^(1,2)^ Although its pathophysiology remains partially understood, FM substantially impairs health-related quality of life (HRQoL) and functional capacity.^(3)^

Diagnostic criteria established by the *American College of Rheumatology (ACR)* have evolved from the 1990 tender-point model to the 2010 and 2016 revisions, which incorporate the *Widespread Pain Index (WPI)* and *Symptom Severity Scale (SSS*), allowing a more comprehensive clinical assessment.^(4-6)^ This condition predominantly affects women of reproductive age, with a global prevalence of 2–8%.^(7,8)^ Hormonal and psychosocial factors appear to modulate this increased susceptibility.^(9)^

Pregnancy and the postpartum period are marked by hormonal, metabolic, and emotional changes that may influence pain perception and exacerbate FM symptoms.^(10,11)^ Preliminary studies suggest a potential association between FM and adverse obstetric outcomes, including increased rates of cesarean delivery, preterm birth, and postpartum mood disturbances.^(12,13)^ However, existing evidence remains limited and fragmented.

Given these uncertainties, a comprehensive synthesis of the literature is warranted. This systematic review aims to evaluate the impact of fibromyalgia during pregnancy and the postpartum period, focusing on quality of life, obstetric outcomes, delivery decisions, and breastfeeding practices.

## Methods

This systematic review was conducted in accordance with the methodological standards of the *Cochrane Collaboration*^(14)^ and reported in line with the *Preferred Reporting Items for Systematic Reviews and Meta-Analyses (PRISMA 2020).*^(15)^ The review protocol was prospectively registered in the International *Prospective Register of Systematic Reviews (PROSPERO CRD42024572007)* to ensure methodological rigor, transparency, and reproducibility.

### Research Question and Framework

The research question was formulated using the PECO framework, defined as follows: Population: pregnant people; Exposure: fibromyalgia; Comparison: pregnant people without fibromyalgia; and Outcomes: quality of life, breastfeeding practices, adverse obstetric and postpartum outcomes. Based on this structure, the central research question was: *"What is the impact of fibromyalgia on quality of life, obstetric complications, and perinatal outcomes in pregnant and postpartum women?"* This formulation guided all stages of study selection and data analysis. This framework guided eligibility criteria, data extraction, and synthesis procedures.

### Search strategy

A comprehensive and systematic search was conducted in the *PubMed/MEDLINE, Cochrane Central Register of Controlled Trials (CENTRAL), EMBASE,* and *SciELO* databases. The search strategy used combinations of Medical Subject Headings (*MeSH*) and *Emtree* terms, as well as free-text keywords related to *"fibromyalgia," "pregnancy," "postpartum," "quality of life,"* and *"treatment."* The complete strategy for each database included *Boolean* operators and controlled vocabulary adapted to each indexing system (e.g., PubMed: ((*"Postpartum Period"(Mesh)) OR "Pregnancy"(Mesh) OR "Pregnant People"(Mesh)) AND "Fibromyalgia"(Mesh))). The specific strategy in each base data is reported in Table 1S. The search covered studies published between January 2010 and May 2024, as 2010 marks the introduction of the revised diagnostic criteria for fibromyalgia. No restrictions were applied regarding publication status. Only studies published in English, Spanish, or Portuguese were considered eligible. The final search was completed in May 2024. All retrieved records were imported into *EndNote 20 (Clarivate Analytics)* for deduplication prior to screening.

### Eligibility criteria

The inclusion criteria encompassed randomized controlled trials, prospective and retrospective cohort studies, and analytical observational designs (case–control or cohort studies) that investigated the effects of fibromyalgia during pregnancy or the postpartum period. Eligible studies evaluated maternal and neonatal outcomes, such as obstetric complications, breastfeeding practices, and quality of life. Studies addressing neuroendocrine mechanisms, chronic pain modulation during pregnancy, or associations between fibromyalgia and adverse maternal-fetal outcomes were also considered relevant. Exclusion criteria included studies involving participants without a confirmed diagnosis of fibromyalgia during pregnancy, those with significant comorbidities (including endocrine, metabolic, or psychiatric disorders), as well as case reports, case series, editorials, or narrative reviews. Only studies providing sufficient methodological detail to permit data extraction and risk-of-bias assessment were included.

### Study selection process

Study selection was independently performed by two reviewers using a two-stage process. First, titles and abstracts retrieved from the databases were screened according to the predefined eligibility criteria. In cases of disagreement, a third reviewer was consulted to resolve discrepancies. Articles deemed potentially relevant were read in full to confirm eligibility. The entire selection process was managed using the *Rayyan web application,*^(16)^ which enables blinded and independent screening. The final set of included studies was determined through consensus among all reviewers. A *PRISMA* 2020 flow diagram was constructed to depict the study selection process.

### Data extraction

Data extraction was performed independently and in duplicate by two reviewers using a standardized data extraction form designed to ensure consistency and minimize bias. The extracted information included the first author, year of publication, country of origin, study design, population characteristics, interventions or exposures, assessed outcomes, and main findings. When necessary, corresponding authors were contacted to clarify or obtain missing data.

### Bias risk assessment

The methodological quality and risk of bias of the included studies were assessed using the Risk of Bias in Non-randomized Studies of Interventions (ROBINS-I) tool.^(17)^ was used, which evaluates potential biases related to confounding, participant selection, exposure classification, outcome measurement, and selective reporting. Disagreements between reviewers were resolved by consensus or with the participation of a senior reviewer (LMTAA). The overall risk of bias was synthesized narratively across studies and visual representations of the risk-of-bias assessment were generated using the *ROBVIS (Risk of Bias VISualization) web tool*.^(18)^

### Data synthesis

Given the heterogeneity among the included studies regarding design, exposure definitions, and outcomes, a qualitative synthesis of the evidence was conducted. The synthesis described and compared the main findings across studies, highlighting convergent and divergent results, methodological limitations, and knowledge gaps. A meta-analysis was not feasible due to the substantial heterogeneity in study populations, outcome definitions, and measurement instruments. Findings were narratively summarized, emphasizing consistency, discrepancies, and methodological strengths and limitations.

## Results

The systematic search of *PubMed, EMBASE, CENTRAL, and SciELO* identified 152 records. After removal of six duplicates, 146 records were screened by title and abstract, leading to the exclusion of 130 studies. Sixteen full-text articles were assessed for eligibility, of which five met the inclusion criteria and were included in the qualitative synthesis (Figure 1). Eleven full-text studies were excluded, mainly due to the absence of confirmed FM diagnosis or insufficient outcome data.

**Figure 1:**
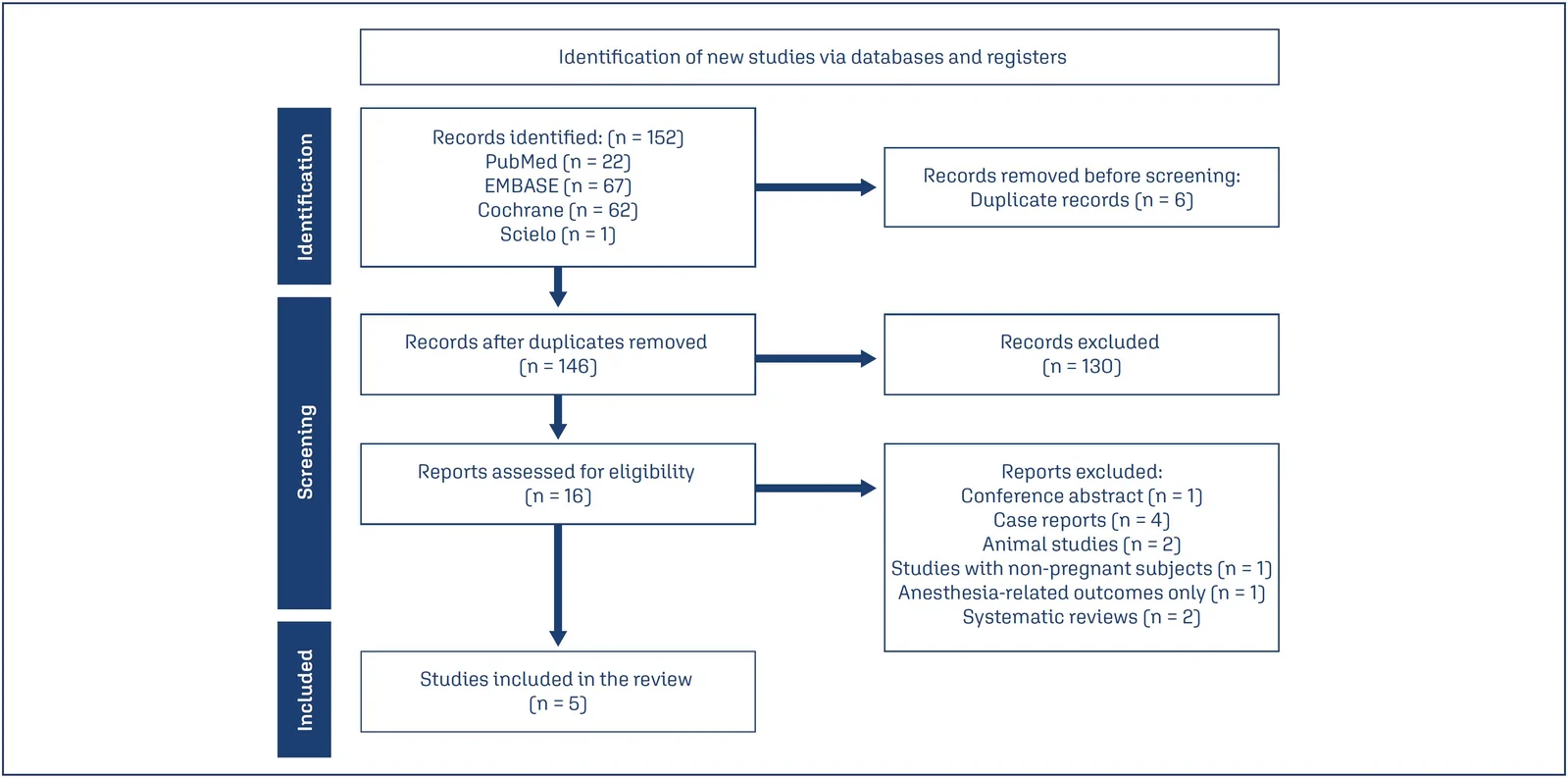
Study selection process.

### Study characteristics

The five included studies,^(12,13,19-21)^ published between 2013 and 2019, employed diverse methodological designs and were conducted in Turkey (n = 3), ^(12,19,21)^ Israel (n = 1),^(13)^ and the United States (n = 1).^(20)^ Collectively, they evaluated 8,182 women diagnosed with FM, encompassing heterogeneous methodological designs. The included studies comprised one large population-based retrospective cohort,^(20)^ two prospective comparative investigations, ^(19,21)^ and two cross-sectional observational studies. ^(12,13)^

Most studies applied the *American College of Rheumatology (ACR)* 2010 or modified 2011 diagnostic criteria enhancing diagnostic comparability across samples.^(12,13,19,21)^ The population-based study by *Magtanong et al.*^(20)^ identified FM cases using the *International Classification of Diseases, Ninth Revision (ICD-9)* coding system.

The included studies investigated a broad range of outcomes spanning obstetric, neonatal, and psychosocial domains. Obstetric and neonatal outcomes, most comprehensively reported by *Magtanong et al. (2019)*, included gestational diabetes, cesarean delivery, preterm birth, placental abruption, venous thromboembolism, and intrauterine growth restriction (IUGR).^(20)^ Psychological and functional outcomes were assessed across the remaining studies using validated instruments such as the *Fibromyalgia Impact Questionnaire (FIQ),*^(12,13,19)^ *Beck Depression Inventory (BDI),*^(12,19,21)^ *State-Trait Anxiety Inventory (STAI)* and *Wijma Delivery Expectancy/Experience Questionnaire (W-DEQ)*^(12)^ and the *Visual Analog Scale (VAS).* ^(12,13,19)^

Three studies investigated women during pregnancy,^(12,19,20)^ while one specifically evaluated women at term,^(13)^ and another included non-pregnant women with FM to retrospectively assess reproductive history and breastfeeding duration.^(21)^ Despite differences in population profiles and methodological designs, the included studies consistently aimed to elucidate the impact of fibromyalgia on maternal health, psychological well-being, and pregnancy-related outcomes. Detailed characteristics of the included studies are summarized in chart 1.

**Chart 1:** Characteristics of the included studies

Author, Year	Country	Study Design	Population (n)	Diagnostic Criteria for FM	Assessment Instruments and Main Outcomes
Genç et al., 2017^(12)^	Turkey	Cross-sectional comparative study	360 pregnant women (136 FM; 224 controls)	ACR 2010	*Clinical:* FIQ, STAI, W-DEQ (fear of childbirth), BDI, VAS.
Saa'd et al., 2013^(13)^	Israel	Cross-sectional observational study	100 term pregnancies (27 meeting FM criteria; 73 controls)	ACR 2011 (modified from 2010)	*Clinical:* labor duration, pain intensity (VAS), quality of life (FIQ, SF-36).
Atasever et al., 2016^(19)^	Turkey	Prospective case–control study	277 pregnant women (150 FM; 127 controls)	ACR 2010	*Biochemical:* serum serotonin levels. *Clinical:* FIQ, WPI, SS Scale, BDI, VAS.
Magtanong et al., 2019^(20)^	United States	Population-based retrospective cohort study	12,584,918 deliveries (7,758 FM)	ICD-9 (code 729.1)	*Maternal outcomes:* gestational diabetes, placental abruption, cesarean delivery, venous thromboembolism. *Neonatal outcomes:* preterm birth, intrauterine growth restriction (IUGR).
Tulay et al., 2016^(21)^	Turkey	Prospective comparative study (non-pregnant women)	187 women (111 FM; 76 controls)	ACR 2010	*Reproductive history:* pregnancy outcomes, parity, breastfeeding duration. *Clinical:* BDI.

ACR - American College of Rheumatology; BDI - Beck Depression Inventory; FIQ - Fibromyalgia Impact Questionnaire; FM - Fibromyalgia; ICD-9 - International Classification of Diseases, Ninth Revision; IUGR - Intrauterine Growth Restriction; SS - Symptom Severity Scale; STAI - State-Trait Anxiety Inventory; VAS - Visual Analog Scale; W-DEQ - Wijma Delivery Expectancy/Experience Questionnaire; WPI - Widespread Pain Index

### Synthesis of results

#### Clinical and psychological outcomes

##### Pain and functional impairment

Across all included studies, pregnant women with FM consistently reported greater pain intensity and reduced functional capacity than healthy controls. In *Atasever et al.*^(19)^., the extent of pain assessed by the *Widespread Pain Index (WPI)* was markedly higher among women with FM (median 7) than in controls (median 1). Similarly, *Symptom Severity Scale (SSS)* scores were substantially elevated (median 6 vs. 0).^(19)^ Two studies^(12,19)^ reported significantly higher pain intensity on the *Visual Analog Scale (VAS)* (median 6 vs. 1, *p* < 0.001 for both). This increased pain burden was directly associated with worse functional outcomes. Both *Atasever et al.*^(19)^ and *Genç et al.*^(12)^ demonstrated significantly higher *FIQ* scores in FM groups compared with controls (median 65–72 vs. 27–29, *p* < 0.001 for all comparisons).^(12,19)^ In *Saa'd et al.*^(13)^ physical functioning assessed by the *SF-36 Health Survey* was also impaired in women meeting criteria for FM (mean 46.85 vs. 65.48, *p* = 0.0003). Notably, although chronic pain perception was markedly heightened, *Saa'd et al.*^(13)^ found no significant correlation between FM symptoms and the intensity of acute pain during labor.

##### Psychological and emotional outcomes

This physical and functional impairment was accompanied by a substantial psychological burden. Across all studies assessing this outcome, women with FM consistently exhibited a higher prevalence and greater severity of depressive symptoms compared to controls. *Atasever et al.*^(19)^ and *Genç et al.*^(12)^ reported significantly higher *BDI* scores in women with FM (median 14–18) compared with controls (median 5–6, *p* < 0.001).^(12,19)^ In the prospective study by *Atasever et al.* ^(19)^, each one-point increase in BDI score was associated with a 1.22-fold higher likelihood of FM (95% CI: 1.16–1.29).^(19)^ Beyond depression, *Genç et al.* ^(12)^ demonstrated that both state and trait anxiety levels evaluated by the State-Trait Anxiety Inventory (STAI) were significantly higher in pregnant women with FM.^(12)^ State scores revealed median state values of 52 versus 25, and trait values of 49 versus 22 (*p* < 0.001 for both). The same study also found that fear of childbirth, assessed by the *W-DEQ*, was significantly more pronounced in women with FM (median 89 vs. 32, *p* < 0.001).^(12)^

#### Obstetric and neonatal outcomes

##### Maternal outcomes

Evidence from the largest population-based cohort indicates that FM appears to be associated with an increased risk of several adverse maternal outcomes. In *Magtanong et al*.^(20)^, women with FM had higher odds of developing gestational diabetes (*adjusted odds ratio (aOR)* 1.16, 95% CI: 1.07–1.27), placental abruption (aOR 1.44, 95% CI: 1.20–1.72), and venous thromboembolism (aOR 2.34, 95% CI: 1.91–2.86).^(20)^ The same study reported a modest yet significant increase in cesarean delivery rates (aOR 1.11, 95% CI: 1.05–1.16).^(20)^ No significant differences were observed for preeclampsia or maternal mortality.^(20)^ Regarding labor characteristics, *Saa'd et al.*^(13)^ identified a significant positive correlation between higher *Fibromyalgia Impact Questionnaire (FIQ)* scores and longer duration of the second stage of labor (*p* < 0.05).^(13)^ Although women with FM showed a higher frequency of epidural anesthesia use compared with controls (87.5% vs. 68.9%), this difference did not reach statistical significance (*p* = 0.10).^(13)^

##### Neonatal outcomes

Maternal FM was also associated with adverse neonatal outcomes. In the large population-based study by *Magtanong et al*.^(20)^, infants born to mothers with FM had significantly higher odds of preterm birth (<37 weeks) (aOR 1.35, 95% CI: 1.25–1.46) and IUGR (aOR 1.48, 95% CI: 1.30–1.68).^(20)^ No significant difference was observed in intrauterine fetal death rates between groups.^(20)^

##### Reproductive history and breastfeeding

One study specifically examined the impact of FM on reproductive history and breastfeeding outcomes by retrospectively assessing a cohort of post-reproductive cohort of non-pregnant women.^(21)^ In this comparative analysis by *Tulay et al.* ^(21)^, no statistically significant differences were observed in gravidity (mean number of pregnancies) or parity (mean number of live births) between women with FM and healthy controls (*p* = 0.252 and *p* = 0.093, respectively).^(21)^ The duration of breastfeeding was also comparable between groups, with a mean duration of 36.20 months in women with FM versus 31.52 months in controls (*p* = 0.075).^(21)^ Despite higher depressive symptom scores among women with FM, no significant differences were observed in reproductive performance or breastfeeding duration.^(21)^

##### Biochemical findings

One prospective case–control study investigated the potential biochemical correlates of FM during pregnancy, with a particular focus on serum serotonin levels.^(19)^ In unadjusted analyses, pregnant women with FM exhibited significantly lower median serotonin levels (32.35 ng/mL) compared with healthy controls (42.15 ng/mL, *p* = 0.009). However, when controlling for potential confounders in a multivariate logistic regression model, serum serotonin was not identified as an independent predictor of FM presence (*p* = 0.549).^(19)^

A weak negative correlation was also observed between serotonin concentrations and *BDI* scores, although this association did not reach statistical significance (*p* = 0.090).^(19)^ No additional biochemical or hormonal abnormalities were identified between the FM and control groups for parameters including fasting glucose, serum calcium, thyroid-stimulating hormone (TSH), and free thyroxine (fT4) levels. These findings suggest that while serotonergic dysregulation may be present in FM, its clinical relevance during pregnancy remains uncertain.^(19)^

##### Risk of bias assessment

The risk of bias was evaluated using the ROBINS-I tool. Four studies^(12,19-21)^ showed a *moderate* overall risk of bias, mainly due to incomplete adjustment for confounders, self-reported outcomes, and limited information on data management. One study^(13)^ was rated *serious* risk, primarily for unadjusted confounding and small sample size.

Selection bias was generally moderate, except for the population-based cohort by *Magtanong et al*.^(20)^ which was low risk due to its registry-based design. Exposure classification was low risk in studies using ACR 2010/2011 diagnostic criteria^(12,13,19,21)^ and moderate in the ICD-coded study.^(20)^ All studies^(12,13,19-21)^ were low risk for deviations from intended interventions.

Bias due to missing data, outcome measurement, and selective reporting was mostly moderate across studies.^(12,13,19,21)^ Overall, the included studies demonstrated acceptable methodological quality, with serious bias limited to a single investigation.^(13)^Figure 2 summarizes domain-level judgments, and Figure 3 shows the distribution of risk levels across ROBINS-I domains.

**Figure 2:**
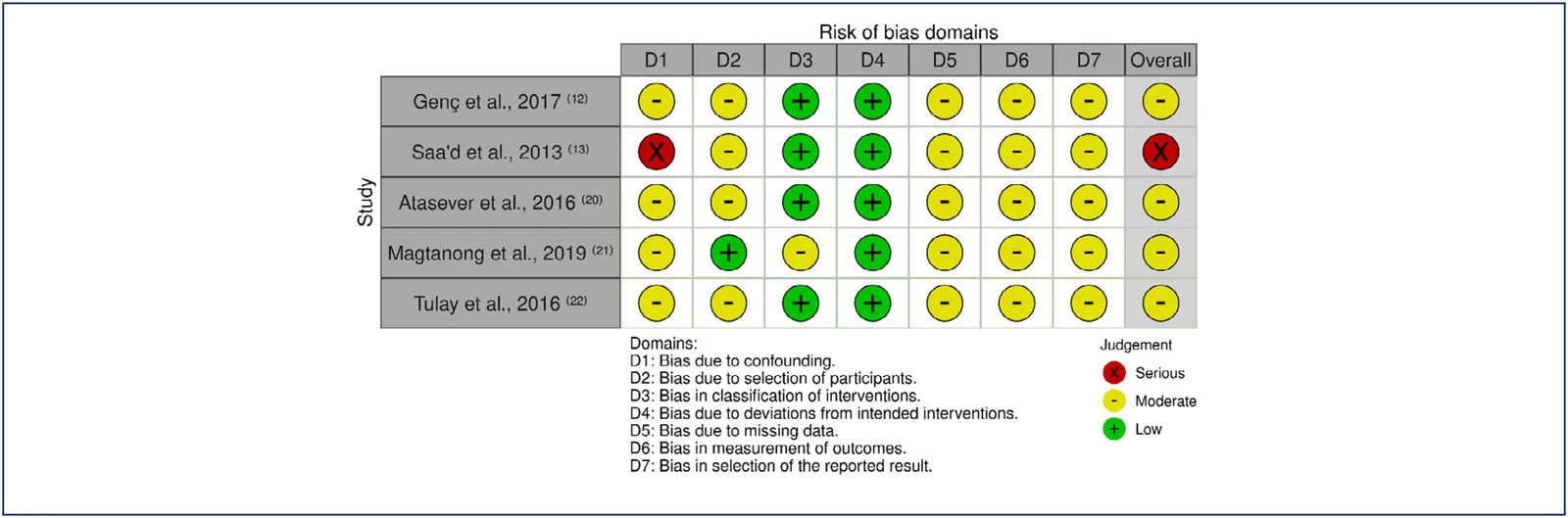
Traffic-light plot showing domain-level ROBINS-I risk-of-bias judgments for the five included studies.

**Figure 3:**
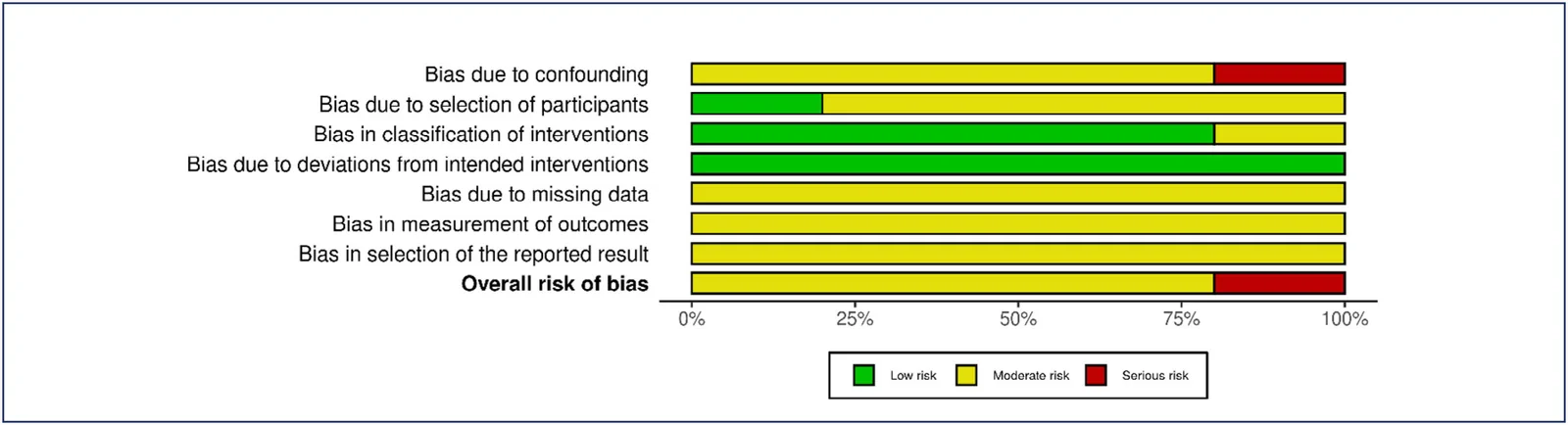
Summary plot depicting the proportion of studies rated at each risk-of-bias level across ROBINS-I domains.

## Discussion

This systematic review demonstrates that FM during pregnancy is consistently associated with reduced HRQoL, greater pain intensity, and higher levels of depressive and anxiety symptoms compared with healthy controls. These findings highlight the substantial psychosomatic and functional burden experienced by affected women.

The population-based cohort by *Magtanong et al.*^(20)^ identified an increased risk of gestational diabetes, cesarean delivery, preterm birth, and IUGR in women with FM. Conversely, *Tulay et al.* ^(21)^ found no significant differences in gravidity, parity, or breastfeeding duration between FM and control groups. Collectively, these findings suggest that while FM adversely affects maternal well-being and psychological status, its direct influence on reproductive performance appears limited.

The observed clinical patterns are consistent with current models describing FM as a neuroimmune and endocrine disorder characterized by altered central pain processing, dysautonomia, and low-grade systemic inflammation.^(22-24)^ Dysregulation of serotonergic transmission, hypothalamic–pituitary–adrenal (HPA) axis imbalance, and persistent sympathetic activation contribute to pain amplification, fatigue, and mood disturbances.^(25,26)^ These mechanisms may become further destabilized during pregnancy, a period of pronounced hormonal and immunological adaptation, potentially intensifying FM symptoms and psychological vulnerability.^(26)^

Moreover, the strong association between FM and depressive or anxiety symptoms aligns with neurobiological evidence of shared serotonergic and inflammatory pathways linking chronic pain and mood disorders. Reduced serotonin bioavailability and elevated proinflammatory cytokines, particularly interleukin (IL)-6 and IL-8, are central mediators of this bidirectional relationship,^(23,25)^ This neuroimmune imbalance likely underpins the heightened emotional distress and pain hypersensitivity reported by pregnant women with FM.

The moderate associations between FM and obstetric complications, such as gestational diabetes, cesarean delivery, and preterm birth,^(20)^ may reflect systemic effects of chronic sympathetic activation, oxidative stress, and endothelial dysfunction, all recognized features of FM pathophysiology.^(24,27,28)^ Although these findings suggest a plausible mechanistic link between FM and adverse pregnancy outcomes, the magnitude of risk remains modest, and residual confounding cannot be excluded.

Furthermore, the observed associations between fibromyalgia and adverse obstetric outcomes should be interpreted with caution. Women with FM frequently present comorbid autoimmune or inflammatory disorders, such as systemic lupus erythematosus or rheumatoid arthritis, which independently increase the risk of gestational complications.^(29,30)^ The available population-based studies did not consistently adjust for these conditions, making it difficult to determine whether the observed excess risk reflects a direct effect of FM pathophysiology or is mediated by concomitant autoimmune processes.

In contrast, the absence of significant differences in fertility indicators and breastfeeding duration supports the interpretation that FM does not directly impair reproductive capacity or postpartum recovery.^(21)^ Its predominant impact appears to occur through psychosocial and neuroendocrine pathways, influencing maternal well-being more than obstetric physiology.^(22,26)^

Regarding pharmacological management, treatment of fibromyalgia during pregnancy requires careful and individualized risk–benefit assessment. Available evidence on duloxetine suggests that pregnancy outcomes may be broadly comparable to those of the general population.^(31)^ However, data remains limited and potentially confounded by underlying maternal conditions.^(31)^ In contrast, recent systematic reviews indicate that pregabalin exposure may be associated with adverse pregnancy and childhood outcomes, warranting additional caution.^(32)^ At the same time, discontinuation of pharmacological therapy may contribute to worsening maternal pain and psychological distress. Therefore, treatment decisions should be individualized, with preference given to non-pharmacological strategies whenever feasible within a multidisciplinary prenatal care approach.

This review has several limitations, as all included evidence is observational, precluding causal inference and the assessment of temporal changes in FM symptoms during pregnancy. Most studies had small sample sizes, particularly those evaluating psychological outcomes, which may reduce statistical power and external validity. Although diagnostic criteria were generally consistent, reliance on ICD-9 coding in one cohort may have introduced misclassification bias. In addition, outcome assessment relied largely on self-reported questionnaires, increasing the risk of recall and measurement bias, and the predominance of single-center studies from Turkey further limits generalizability.

Importantly, these methodological limitations reduce the overall certainty of the evidence. As indicated by the risk-of-bias assessment, one study presented a serious risk of bias, while the remaining studies showed moderate risk, mainly related to residual confounding and self-reported outcomes. Therefore, the observed associations should be interpreted with caution.

Clinically, FM during pregnancy appears to be associated with increased pain, functional impairment, and psychological distress, underscoring the need for integrated obstetric, psychological, and pain management strategies. Routine screening for depression and anxiety should be considered in prenatal care, and non-pharmacological interventions such as cognitive-behavioral therapy, physical activity, and sleep optimization should be prioritized.

## Conclusion

Fibromyalgia during pregnancy appears to be associated with greater pain, functional impairment, and increased depressive and anxiety symptoms, adversely affecting maternal HRQoL. Evidence also suggests a modest increase in selected obstetric risks, while reproductive capacity and breastfeeding outcomes seem largely preserved. Given the observational nature and methodological limitations of the available evidence, these findings should be interpreted with caution. Multidisciplinary prenatal care focusing on psychological support and non-pharmacological pain management is recommended, and further well-designed prospective studies are needed to strengthen the evidence base.

## Data Availability

The research data are described in the article presented.

## References

[1] Clauw DJ (2014). Fibromyalgia. JAMA.

[2] Häuser W, Ablin J, Fitzcharles MA, Littlejohn G, Luciano J V., Usui C (2015). Fibromyalgia. Nat Rev Dis Primers.

[3] Bennett RM, Friend R, Jones KD, Ward R, Han BK, Ross RL (2009). The Revised Fibromyalgia Impact Questionnaire (FIQR): validation and psychometric properties. Arthritis Res Ther.

[4] Wolfe F, Clauw DJ, Fitzcharles M, Goldenberg DL, Katz RS, Mease P (2010). The American College of Rheumatology Preliminary Diagnostic Criteria for Fibromyalgia and Measurement of Symptom Severity. Arthritis Care Res (Hoboken).

[5] Wolfe F, Smythe HA, Yunus MB, Bennett RM, Bombardier C, Goldenberg DL (1990). The american college of rheumatology 1990 criteria for the classification of fibromyalgia. Arthritis Rheum.

[6] Wolfe F, Clauw DJ, Fitzcharles MA, Goldenberg DL, Häuser W, Katz RL (2016). 2016 Revisions to the 2010/2011 fibromyalgia diagnostic criteria. Semin Arthritis Rheum.

[7] Queiroz LP (2013). Worldwide Epidemiology of Fibromyalgia. Curr Pain Headache Rep.

[8] Marques AP, Santo A de S do E, Berssaneti AA, Matsutani LA, Yuan SLK (2017). Prevalence of fibromyalgia: literature review update. Revista Brasileira de Reumatologia (English Edition).

[9] Ablin JN, Buskila D (2013). Fibromyalgia syndrome – Novel therapeutic targets. Maturitas.

[10] Malviya AK, Gupta A, Kumar V, Gupta J, Deepika, Khanna P (2025). Effect of pregnancy hormones on pain perception in the peripartum period: A narrative review. J Anaesthesiol Clin Pharmacol.

[11] Schaefer CP, Adams EH, Udall M, Masters ET, Mann RM, Daniel SR (2016). Fibromyalgia Outcomes Over Time: Results from a Prospective Observational Study in the United States. Open Rheumatol J.

[12] Genç H (2017). The Effects of Fibromyalgia Syndrome on Physical Function and Psychological Status of Pregnant Females. Arch Rheumatol.

[13] Saa'd S, Many A, Jacob G, Ablin JN (2013). High prevalence of fibromyalgia symptoms among healthy full-term pregnant women. Rheumatol Int.

[14] Higgins JPT, Thomas James, Chandler Jackie, Cumpston Miranda, Li Tianjing, Page MJ (2020). Cochrane handbook for systematic reviews of interventions. Wiley-Blackwell.

[15] Page MJ, McKenzie JE, Bossuyt PM, Boutron I, Hoffmann TC, Mulrow CD (2021). The PRISMA 2020 statement: an updated guideline for reporting systematic reviews. BMJ.

[16] Ouzzani M, Hammady H, Fedorowicz Z, Elmagarmid A (2016). Rayyan—a web and mobile app for systematic reviews. Syst Rev.

[17] Sterne JA, Hernán MA, Reeves BC, Savović J, Berkman ND, Viswanathan M (2016). ROBINS-I: a tool for assessing risk of bias in non-randomised studies of interventions. BMJ.

[18] McGuinness LA, Higgins JPT (2021). Risk-of-bias VISualization (robvis): An R package and Shiny web app for visualizing risk-of-bias assessments. Res Synth Methods.

[19] Atasever M, Namlı Kalem M, Sönmez Ç, Seval MM, Yüce T, Sahin Aker S (2017). Lower serotonin level and higher rate of fibromyalgia syndrome with advancing pregnancy. The Journal of Maternal-Fetal & Neonatal Medicine.

[20] Magtanong GG, Spence AR, Czuzoj-Shulman N, Abenhaim HA (2019). Maternal and neonatal outcomes among pregnant women with fibromyalgia: a population-based study of 12 million births. The Journal of Maternal-Fetal & Neonatal Medicine.

[21] Tulay KT, Tanrıkut E, Arslan A, Özdemir F (2016). The effect of fibromyalgia syndrome to gravidity, parity and duration of breastfeeding; A prospective study from Turkey. Pak J Med Sci.

[22] Ablin JN, Buskila D (2015). Update on the genetics of the fibromyalgia syndrome. Best Practice and Research: Clinical Rheumatology. Bailliere Tindall Ltd.

[23] Kosek E, Altawil R, Kadetoff D, Finn A, Westman M, Le Maître E (2015). Evidence of different mediators of central inflammation in dysfunctional and inflammatory pain - Interleukin-8 in fibromyalgia and interleukin-1 β in rheumatoid arthritis. J Neuroimmunol.

[24] Häuser W, Sarzi-Puttini P, Fitzcharles MA (2019). Fibromyalgia syndrome: under-, over- and misdiagnosis. Clin Exp Rheumatol.

[25] Loçasso FA, Filho HA, Alvarenga RM, Schimidt SL, Fiorelli FK, Ramos PD (2024). Assessing the impact of IL-6 and serotonin on Pain and symptomatology in fibromyalgia: an exploratory clinical study. J Pers Med.

[26] Mucci V, Demori I, Browne CJ, Deblieck C, Burlando B (2023). Fibromyalgia in Pregnancy: Neuro-Endocrine Fluctuations Provide Insight into Pathophysiology and Neuromodulation Treatment. Biomedicines.

[27] El-Sawy N, El-Tantawi G, Achmawi GAH, Sultan H, Younis S (2012). Autonomic changes in fibromyalgia: Clinical and electrophysiological study. Alexandria Journal of Medicine.

[28] Kulshreshtha P, Deepak KK (2013). Autonomic nervous system profile in fibromyalgia patients and its modulation by exercise: a mini review. Clin Physiol Funct Imaging.

[29] Andreoli L, Gerardi MC, Fernandes M, Bortoluzzi A, Bellando-Randone S, Brucato A (2019). Disease activity assessment of rheumatic diseases during pregnancy: a comprehensive review of indices used in clinical studies. Autoimmun Rev.

[30] Buyon JP, Kim MY, Guerra MM, Laskin CA, Petri M, Lockshin MD (2015). Predictors of Pregnancy Outcomes in Patients With Lupus: A Cohort Study. Ann Intern Med.

[31] Hoog SL, Cheng Y, Elpers J, Dowsett SA (2013). Duloxetine and Pregnancy Outcomes: Safety Surveillance Findings. Int J Med Sci.

[32] Beau AB, Mo J, Moisset X, Bénévent J, Damase-Michel C (2025). Systematic review of gabapentinoid use during pregnancy and its impact on pregnancy and childhood outcomes: A ConcePTION study. Therapies.

