# Supplementary material for: Fibromyalgia in pregnancy and postpartum period: effects on quality of life, obstetric outcomes, delivery decisions, and breastfeeding: a systematic review
Source: Rev Bras Ginecol Obstet. 2026 Aug 20;48:e-rbgo49. doi: 10.61622/rbgo/2026rbgo49 (PMC13690381; doi:10.61622/rbgo/2026rbgo49)
Supplement: Supplementary file 1 [file 1806-9339-rbgo-48-e-rbgo49-Supp01.pdf]

Supplementary material

Title: *Fibromyalgia in Pregnancy and Postpartum Period: Effects on Quality of Life, Obstetric Outcomes, Delivery Decisions, and Breastfeeding: A Systematic Review*

Objective of Review

To systematically evaluate the impact of fibromyalgia (FM) during pregnancy and the postpartum period on health-related quality of life *(HRQoL)*, obstetric outcomes, delivery decisions, and breastfeeding practices.

Table 1S. Search strategy

**PubMed**<br>*(("postpartum period"[MeSH Terms] OR "pregnancy"[MeSH Terms] OR "pregnant people"[MeSH Terms] OR "pregnant people"[Title/Abstract] OR "postpartum"[Title/Abstract]) AND ("fibromyalgia"[MeSH Terms] OR "fibromyalgia"[Title/Abstract]) AND ("quality of life"[MeSH Terms] OR "quality of life"[Title/Abstract] OR "therapeutics"[MeSH Terms] OR "treatment"[Title/Abstract]))*
**SciELO**<br>*(pregnancy OR gravidez OR postpartum OR pós-parto OR puerperio) AND (fibromyalgia OR fibromialgia)*
**EMBASE**<br>*('pregnancy'/exp OR 'puerperium'/exp OR 'postpartum period'/exp OR pregnan*:ti,ab OR postpartum:ti,ab) AND ('fibromyalgia'/exp OR fibromyalgia*:ti,ab)*
**Cochrane CENTRAL**<br>*("postpartum period" OR pregnancy OR "pregnant women" OR "pregnant people") AND fibromyalgia*

Search in each database

PubMed
Total number of articles: 22
EMBASE
Total number of articles: 67
Cochrane CENTRAL
Total number of articles: 62
SciELO
Total number of articles: 1
Articles Included
The included articles can be seen in the main manuscript and in the *PRISMA* flowchart.
Total articles found: 152
Total after removing duplicates: 146
Total assessed for eligibility: 16
Excluded: 11 articles (conference abstract = 1; case reports = 4; animal studies = 2; non-pregnant subjects = 1; anesthesia-related = 1; systematic reviews = 2)
Total included in this review: 05 articles
